# Nest Site Selection by Kentish Plover Suggests a Trade-Off between Nest-Crypsis and Predator Detection Strategies

**DOI:** 10.1371/journal.pone.0107121

**Published:** 2014-09-10

**Authors:** Miguel Ángel Gómez-Serrano, Pascual López-López

**Affiliations:** Vertebrates Zoology Research Group, CIBIO Research Institute, University of Alicante, Alicante, Spain; University of Sussex, United Kingdom

## Abstract

Predation is one of the main causes of adult mortality and breeding failure for ground-nesting birds. Micro-habitat structure around nests plays a critical role in minimizing predation risk. Plovers nest in sites with little vegetation cover to maximize the incubating adult visibility, but many studies suggest a trade-off between nest-crypsis and predator detection strategies. However, this trade-off has not been explored in detail because methods used so far do not allow estimating the visibility with regards to critical factors such as slope or plant permeability to vision. Here, we tested the hypothesis that Kentish plovers select exposed sites according to a predator detection strategy, and the hypothesis that more concealed nests survive longer according to a crypsis strategy. To this end, we obtained an accurate estimation of the incubating adult's field of vision through a custom built inverted periscope. Our results showed that plovers selected nest sites with higher visibility than control points randomly selected with regards to humans and dogs, although nests located in sites with higher vegetation cover survived longer. In addition, the flushing distance (i.e., the distance at which incubating adults leave the nest when they detect a potential predator) decreased with vegetation cover. Consequently, the advantages of concealing the nest were limited by the ability to detect predators, thus indirectly supporting the existence of the trade-off between crypsis and predator detection. Finally, human disturbance also constrained nest choice, forcing plovers to move to inland sites that were less suitable because of higher vegetation cover, and modulated flushing behavior, since plovers that were habituated to humans left their nests closer to potential predators. This constraint on the width of suitable breeding habitat is particularly relevant for the conservation of Kentish Plover in sand beaches, especially under the current context of coastal regression and increase of recreational activities.

## Introduction

Predation is the main cause of breeding failure for ground-nesting birds [1] and one of the most important causes of adult mortality, particularly during incubation [2]. Mortality of adults eliminates all future reproductive opportunities and hence, birds have been suggested to maximize lifetime and reproductive success through the achievement of an optimal balance between reproduction and predator avoidance [3]. Thus, birds particularly vulnerable to predation during reproduction, such as ground-nesting species, tend to produce abundant offspring and employ strategies to minimize the risk of adult and egg predation [4], [5], [6].

Predation risk on adults and eggs can be minimized through an adequate nest choice, particularly in ground-nesting species. In fact, micro-habitat structure and the degree of concealment, play a critical role in determining nest fate against predators [2], [7]. Predation avoidance is achieved through two different nesting strategies amongst shorebirds [8]. While some species employ a crypsis strategy based on nesting in habitats with dense and tall vegetation as a way to camouflage clutches against predation [e.g. 9,10], others use a predator detection strategy, based on breeding in open habitats so as to increase the visibility of incubating adults and the early detection of predators [8]. Most studies of nest site selection of plovers show evidence for the second strategy [11]–[15]. Nevertheless, other studies suggest a trade-off between nest crypsis and the ability of incubating adults to detect predators [7], [16]–[18].

Kentish Plover (*Charadrius alexandrinus*) may breed in different habitats throughout its range, including coastal beaches, river gravel and sand bars, salt pans, and salt flats [11], [19], [20]. Despite this apparent plasticity, plovers nest almost exclusively on exposed sites in sparsely vegetated areas [11], [13], [14], [21]–[23].

The relationship between vegetation cover and visibility from nests has received some attention so far [2], [15], [24]. However, the trade-off between nest crypsis and predator detection has not been explored in detail, mainly because the methods used do not allow estimating the actual view of the surroundings from the nest by the incubating adult. In particular, most analyses of nest site selection have been undertaken at small spatial scales (usually ≤1 m from the nest) [13], [25], [26], and have not taken into account, the fact that vegetation may be permeable to vision. However, both aspects (i.e., spatial scale and visual permeability) are critical to assess nest site selection in relation to predation risk.

Here we analyze micro-habitat nest site selection by Kentish Plovers breeding on sandy beaches and examine the influence of vegetation cover on nest survival. To this end, we estimated the visibility of incubating adults with regard to vegetation and ground relief. Specific goals were to examine: (1) whether nest site choice was dependent on visibility, taking into account the detectability of predators; (2) the impact of nest-site selection on nest success; and (3) nest-site selection patterns relative to human disturbance. If a trade-off between nest crypsis and predator detection strategies exist, we predict that nest success should be higher in concealed sites as a result of lower predation rate on eggs and moreover birds should select nest sites with higher visibility than random sites in order to maximize predator detection.

## Materials and Methods

### Study species

Kentish Plover is a ground nesting shorebird distributed along Eurasia and Africa [27]. Recently, European and American populations of the nominal species (*Charadrius alexandrinus*) have been split into two different species, the Kentish Plover in Eurasia and Africa, and the Snowy Plover (*Charadrius nivosus*) in the Americas [28]. Despite the fact that the Kentish Plover is declining, the species is not globally threatened and is listed as Least Concern worldwide according to IUCN red list [27]. It is listed as vulnerable in Spain [29]. At regional level, in our study area, it is listed as a threatened species since 2013. Along the Mediterranean coast of Spain, its population decline is attributed, at least partially, to habitat degradation associated with the increase of human disturbance [19], [29], [30]. Sandy beaches are an important natural breeding habitat for Kentish Plover, but are usually valued by humans for recreation. Human disturbance on sandy beaches may affect breeding success or force birds to nest in alternative habitats [31].

### Ethics statement

Corresponding permissions were granted by the Spanish Regional Administration “Conselleria de Infraestructuras, Territorio y Medio Ambiente” (permit 078/07), and the Devesa-Albufera Service of city council of Valencia facilitated access to the Punta Beach reserve (Albufera Natural Park). According to the Spanish law “Ley 42/2007 de 13 de diciembre del Patrimonio Natural y la Biodiversidad” an ethical approval is not required for this study. This paper complies with the current laws in Spain.

### Study area

**Figure 1:**
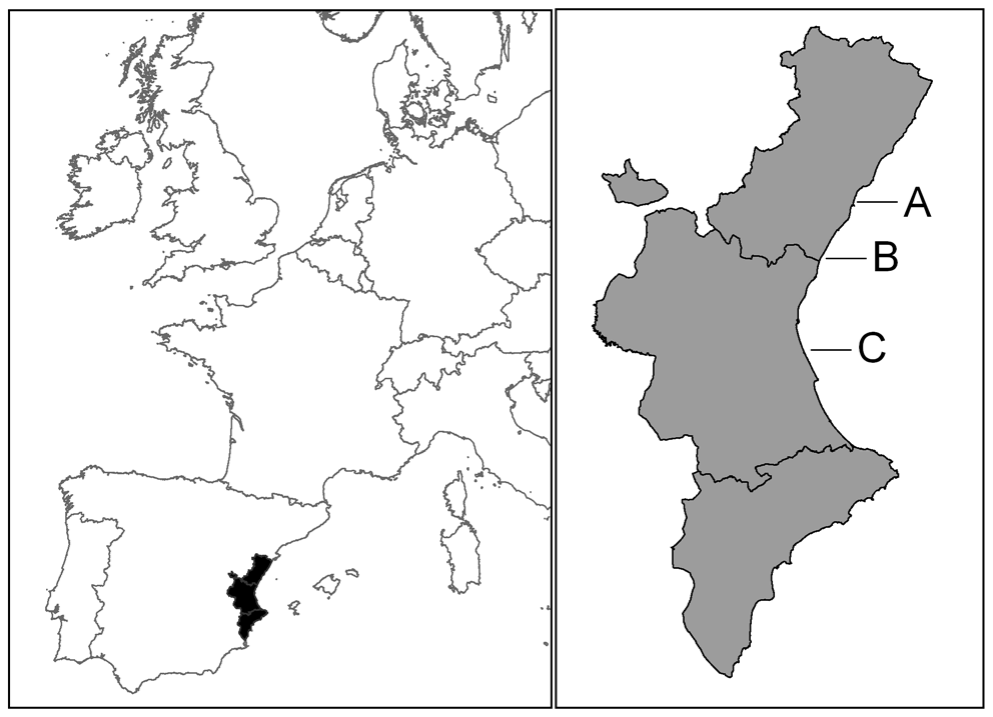
Study area. From left to right, Serradal (A), Almenara (B) and Punta (C) beaches are shown. Upper right inset map shows the Valencian Community in Western Europe. The exact location of the three beaches are shown in the inset map below.

We sampled three beaches in the Castellón and Valencia provinces (Eastern Spain; Fig. 1): Serradal (Castellón de la Plana, 40° 00′ N, 0° 01′ E), Almenara (39° 43′ N, 0° 11′ W) and Punta (Valencia 39° 18′ N, 0° 17′ W). All three beaches have natural dune vegetation. Punta (1.2 km in length) and Serradal (1.1 km) are natural sandy beaches. Almenara (2.3 km in length) is a natural beach of mixed sandy areas with gravel and pebbles. The three beaches benefit from different types of legal protection according to European and regional legislation. In these sites Kentish plovers nest primarily on embryonic shifting dunes and annual vegetation of drift lines, but also in grasslands of small annual plants that grow on deep sand areas among dry interdunal depressions. Dominant species of these habitats include *Elymus farctus*, *Ammophila arenaria*, *Medicago marina*, *Lotus creticus*, *Otanthus maritimus*, *Pancratium maritimum, Sporobolus pungens* and *Cakile maritima*.

The three beaches are subject to a different intensity of human disturbance. Serradal is a beach frequented by people for leisure (>10 people/km/hour; authors' unpubl. data). Almenara has an intermediate level of human disturbance, with lower human presence with regard to Serradal (1–5 people/km/hour; authors' unpubl. data). Finally, Punta is a bird sanctuary with restricted access, where human use is almost negligible (managers and occasionally trespassers).

### Field procedure

This study was conducted during two different periods. Firstly, research was carried out on Serradal between 1992 and 2001 during each breeding season; secondly, between 2007 and 2008 in the three study areas simultaneously. The same observer recorded all data across study areas and years.

Kentish plover nests were located by systematically combing beaches and dune systems on foot from early March to late July. Most clutches were located following the density of plovers' footprints on the sand, generally in sites where incubating adults where seen flushing the nests or displaying distraction behavior (simulation of incubating, potentially injured bird, etc.) in the vicinity of nests.

Once a nest was found, it was individually marked and visited every 3–5 days to measure clutch size and nest fate. There were no differences in the rates of nest visits across years and study sites. We marked each egg so as to identify it during subsequent visits and to record egg-turning activity.

We assessed laying date according to clutch size and laying interval for Kentish Plover [32], [33]. We assumed that nests with one egg had been initiated the same day they were encountered, whereas those with two eggs and a third one observed in the following visit were considered to have been started the day before. Laying date in nests with complete clutches (i.e. with three eggs, the modal clutch size, or two eggs without a third one on a subsequent visit) was estimated using the hatching date or through the egg-flotation pattern [34], [35]. Alternatively, when the laying date was unknown (i.e. the nest was found with complete clutch) but the nest scrape was previously recorded, we assumed that laying date had taken place midway between the last and the following visit.

We considered that nests were active when they were attended by adults for incubation tasks. Evidence of nest activity included: (i) the observation of incubating parents; (ii) the observation of incubating parents flushing from the nest when the observer approached; (iii) the observation of adults performing distraction displays to potential predators (in most cases the observer) within the vicinity of the nest; (iv) egg-turning since the previous visit; (v) normal development according to the egg-flotation scheme [35]; and (vi) a high density of plover footprints in the sand around the nest scrape. We considered that nest was deserted if there was no evidence of the formerly described signs of activity. We assumed that both predation and desertion have occurred midway between the last visit with nest activity and the following visit.

Nests were considered successful when at least one egg hatched. Evidence of hatching included the presence of (i) chicks; (ii) eggshell evidences (i.e. small pieces of detached eggshell membranes in the nest scrape) [35], [36]; (iii) adults with chicks or adults performing distraction displays when nests scrapes were empty close to hatching date. Evidence of predation included (i) partially consumed eggs in the nests scrapes and their surroundings, (ii) the presence of a mixture of yolk and sand from broken eggs, or (iii) the disappearance of eggs before expected hatching date.

For each nest, we calculated survival rate as the number of days elapsed from the laying of the first egg until the hatching of last egg, or until predation or desertion. The average maximum number of days that nests typically survived is 31 [2].

### Habitat type

Each nest was assigned to one of the following habitat types: i) tidal debris (i.e., beach area outside the tidal zone where scattered organic and inorganic remains washed by the sea accumulate; ii) embryonic shifting dunes (i.e., first stages of dune construction, made up of ripples or raised sand bars of the upper parts of the beach); iii) shifting dunes (mobile dunes forming seaward dunes, typically following embryonic shifting dunes); and iv) semi-fixed dunes (i.e., dunes with little relief at the rear of shifting dunes, characterized by a vegetation dominated by bulbous plants and small sized scrubs). The latter habitat type includes nests located in grasslands of small annual plants that grow on deep sand areas among dry interdunal depressions.

### Vegetation cover

We assessed the degree of vegetation cover within a one meter size square centered on the nest. For this purpose, we used Munsell Soil Charts [37] for estimating proportions of mottles and coarse fragments. These charts allow visual estimation of the relative cover of fragments (in our case vegetation patches) within squares according to the following percentages: 0, 1, 2, 3, 5, 7, 10, 15, 20, 25, 30, 40, 50 and >50%.

### Visibility

**Figure 2:**
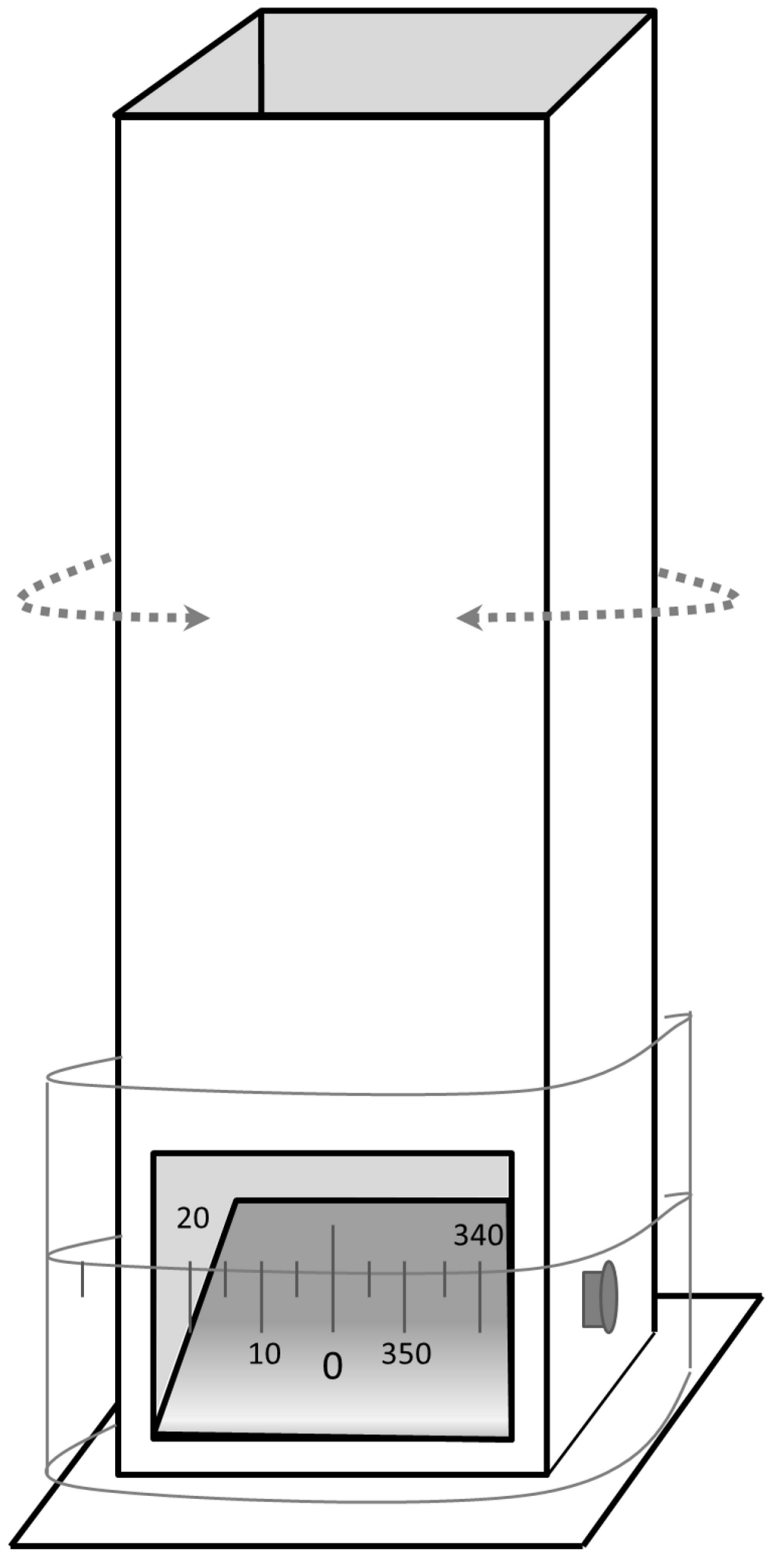
Inverted periscope used for the estimation of birds' visibility from the nest.

During 1999 (Serradal) and 2008 (Punta) we assessed the degree of visibility from the nest to quantify the ability of incubating plovers to detect ground predators. Visibility from nests was measured using a custom built periscope, similar to that designed by [38] for a microhabitat study with larks. The periscope was designed to gain an accurate estimation of the incubating adult's field of vision. It has a movable mirror inside oriented to a window placed at the bottom, just at the height of the bird's-eye view. This allowed estimating the view of the incubating bird to potential predators. A transparent graduated plastic around the periscope allowed the measurement of the sum of degrees of visibility (i.e., from 0° to 360°) to the nearest five degrees (Fig. 2).

We used the periscope to measure the angle of visibility from the nest, and to determine if birds might be able to detect the presence of a person 1.70 m tall standing 25 m around the nest. Then, this person walked 360° around the nest (25 m radius from the nest, using a rope) and the observer recorded the sum of degrees out of the complete circumference that this person was visible from the nest. The same experiment was performed to estimate the detectability of a medium-sized predator (e.g. a dog) of an average height (0.50 m). Predator visibility was estimated using a red ribbon knotted on the person's leg. In order to avoid disturbing incubating birds, we recorded the visibility at each nest scrape just following hatching completion. The growth period of the dune vegetation in the study area occurs in winter (from November to February) and the senescence period starts from July. Taking into account that the laying period of the Kentish plover spans from late March-early April to late June, changes in vegetation cover between nest-site selection and hatching completion were negligible.

For comparisons, we use as controls the same number of points as nests. Control points were obtained for each nest by moving the periscope 10 m in a random direction. We replaced any control point that occurred on a substrate other than sand (for example water or very dense plant cover). We calculated the degree of visibility from control points with the same method as for nests.

We use a 25 m distance because our results of the flushing behavior experiment in one of the study sites revealed that plovers departed from the nest at an average flushing distance of 23.1±12.3 m when the observer approached. Although other studies reported higher flushing distances [2], [13], we considered that 25 m represents a realistic distance for the particular case of our study area.

**Figure 3:**
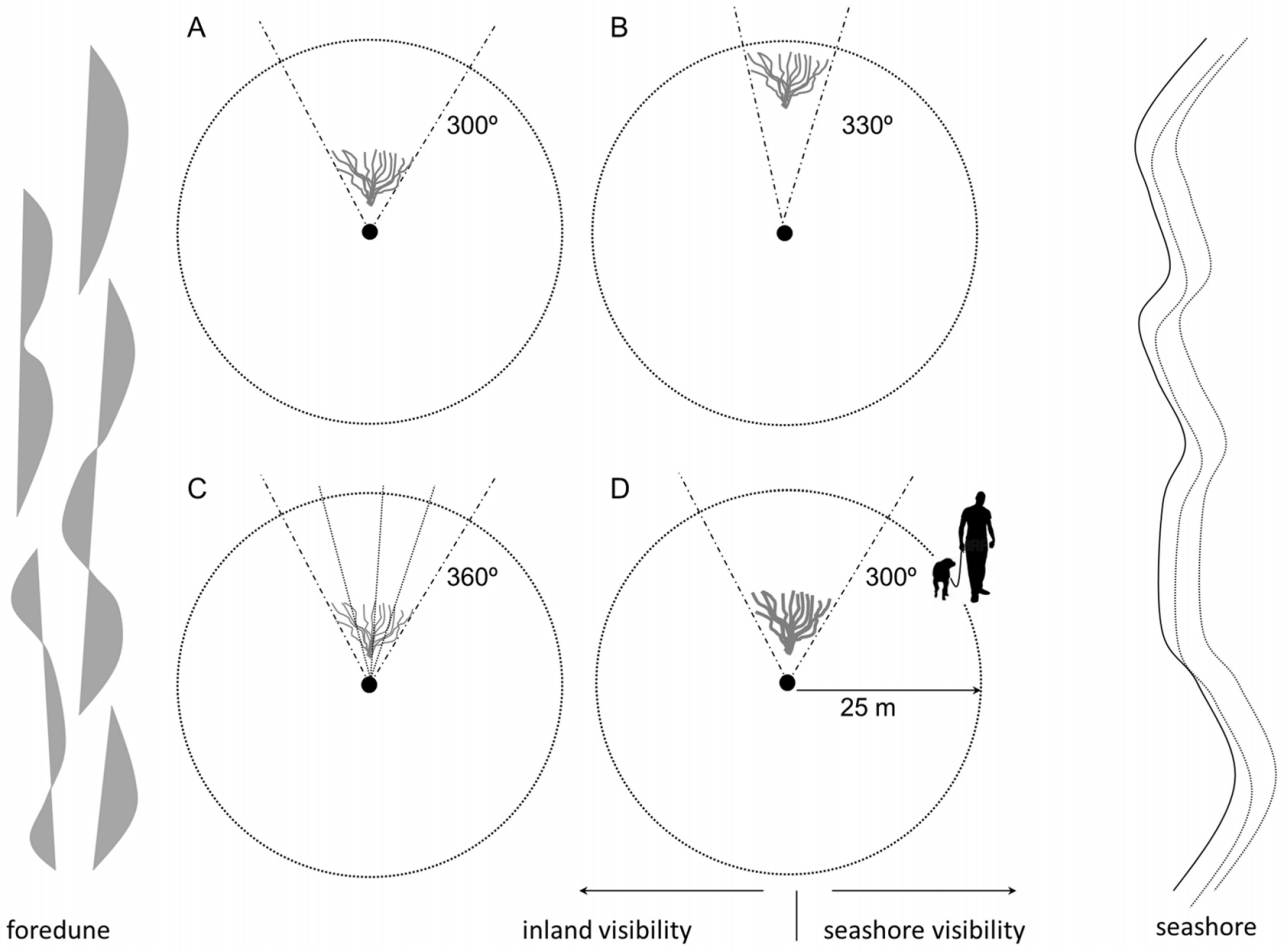
Experimental design to record visibility from Kentish Plovers' nests. Black dots show the location of Kentish plovers' nests. Circular dashed lines shows the perimeter of the circle (25 m radius) in which the visibility of both humans and dogs were recorded. Inland and seashore visibility were recorded separately by dividing the circle in two 180° sectors parallel to the seashore. The figure illustrates four different situations in which vegetation may obstruct plovers' view: a plant close to the nest (A) obstructs more (60° of view occluded) than the same plant farther from the nest (B) (30° of view occluded). In addition, a plant located at the same distance from the nest may allow the visibility through it (C) or may obstruct totally the visibility (D) depending on the permeability of the foliage density.

Potential predators (e.g. dogs, humans) could gain access to the plover's breeding grounds from inland (e.g. adjacent promenades) or the seashore. To explore differences in birds' visibility with regard to the two types of access routes, we recorded for each nest seashore (from 0° to 180°) and inland (from 180° to 360°) visibility, separately (Fig. 3). Taking into account the sparse vegetation cover and the low height of plants around nests in our study area (usually below 25 cm) that very rarely shades the nests, the potential influence of air predators in nest site selection was not assessed (i.e., we considered that there were no limitations in the visibility to potential air predators from nests). The only exception would be birds whose hunting technique consisted in a ground-hugging flight, whose potential risk would be included in the experimental design.

### Flushing behavior

Kentish Plovers rely on eggs' crypsis to conceal their nests [22], [32]. In order to calculate flushing distance (i.e., the distance at which incubating adults leave the nest when they detect a potential predator), and whether this was related to vegetation cover, we conducted an experiment in which an observer walked in a straight line towards the nest. To this end, we walked directly from a distance of 150 m at constant speed in order to avoid bias associated with flush initiation distance [39]. The direction from which the observer approached the nest was randomized. When the incubating adult (usually the female during daytime) left the nest, we scored with a tape measure the distance between the observer and the nest. We only use the data for those cases in which we were able to record visually the precise moment when plovers departed from nests. In addition, to avoid a possible cumulative effect of humans' presence on flushing behavior, we only considered data collected from nests that had not been previously visited by us on the same day, and when humans had not been observed in the vicinity of the nest for at least one hour before the experiment. For the same reason, we did not approach the same nest more than once daily [13].

To account for variations in flushing behavior, we conducted experiments during both morning and afternoon and recorded sand temperature and incubation period (days since the first egg was laid) in each of the approaches to the nests. For analyses, data were grouped according to two categories: morning (8:00h to 12:00h) and afternoon (12:00h to 21:00h).

In addition, we also recorded whether plovers flushed from the nest or remained incubating in relation to two different situations: i) presence of people walking, and ii) people walking unleashed dogs, both at a distance between 25 and 75 m from nests. Observations were conducted between 18:00 and 20:00h in the beach with high level of human disturbance (Serradal). During this period we only recorded the first disturbance event for each nest. Observations for the same nest were separated at least one week. To avoid the cumulative effect of the number of people on nest disturbance we used the events in which one or two people were involved for analyses.

### Statistical Analyses

Statistical analyses were carried out using SPSS v.19 software (SPSS, Chicago, IL, USA) and R package [40]. Descriptive statistics are represented as the mean ± standard deviation. Data were tested for normality before being analyzed with parametric tests with one-sample Kolmogorov-Smirnov test. Comparison of continuous data between nest success and vegetation cover was carried out by using the Mann-Whitney U-test, and of categorical variables by using the Chi-squared (χ^2^) test. Mann-Whitney tests were used to investigate differences between nest visibility and control points. The differences between the visibility of humans and dogs at the same site (nest or control point) were analyzed using the Wilcoxon Signed Ranks Test. Comparison of continuous data of flushing distances was carried out by using an unpaired Student's t-test. Finally, Spearman's correlation coefficient analysis was conducted to assess the relationships between flushing distance and vegetation cover, nest age (i.e., days of incubation) and sand temperature.

We used mixed-effects Cox proportional hazard models to test the effect of vegetation cover, habitat type and its combination on nest survival. This allowed us to deal with experimental design and to include some nests as censored data up to the point of desertion [41]. Since data were sampled in different years and beaches, we included year and beach as random effects in survival models. Mixed-effects Cox models were fitted using the R package “coxme” version 2.2-3 [42] and compared using a likelihood ratio test [41].

## Results

### Habitat type and vegetation cover

**Figure 4:**
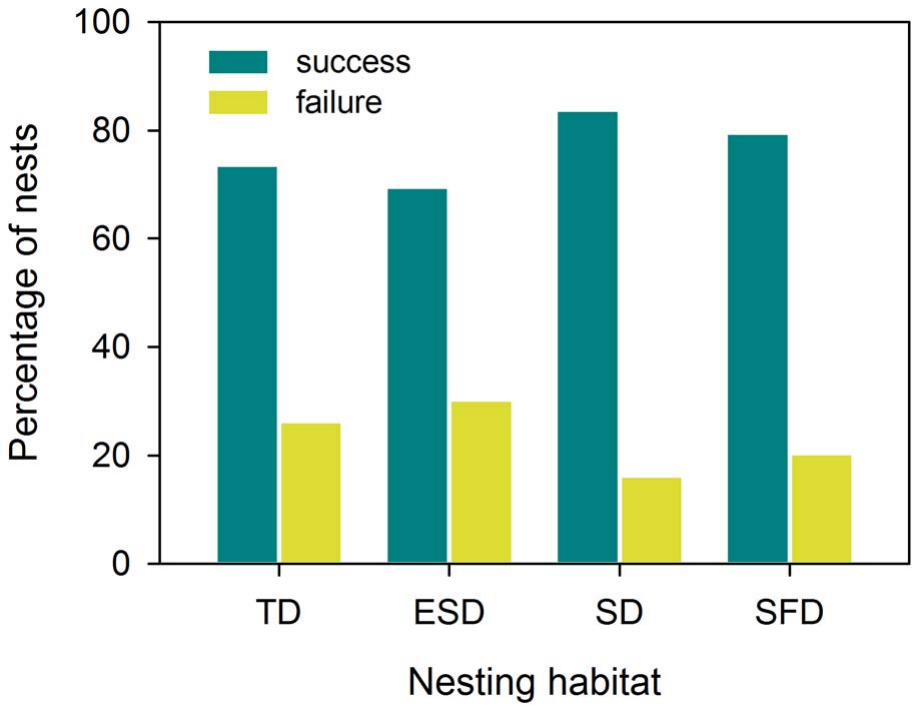
Nest fate in relation to habitat type. From left to right the distance to seashore increases. Abbreviations and sample size: tidal debris (TD; N = 38), embryonic shifting dunes (ESD; N = 56), shifting dunes (SD; N = 80) and semi-fixed dunes (SFD; N = 142).

We analyzed 316 plover nests, of which nest fate was recorded. 38 nests were located in tidal debris, 56 in embryonic shifting dunes, 80 in shifting dunes and 142 in semi-fixed dunes. Nest failure was higher in dune habitats closer to the sea (Fig. 4). Nests on tidal debris and embryonic shifting dunes had higher failure rates (26.32% and 30.36% respectively) than those that were located in shifting dunes and semi-fixed dunes (16.25% and 20.42%, respectively). Differences in breeding success were significant when comparing the most exposed habitats to predators (i.e., tidal debris and embryonic shifting dunes) with those less exposed (i.e., shifting dunes and semi-fixed dunes) (χ^2^ = 3.999, d.f. = 1, P<0.046).

**Figure 5:**
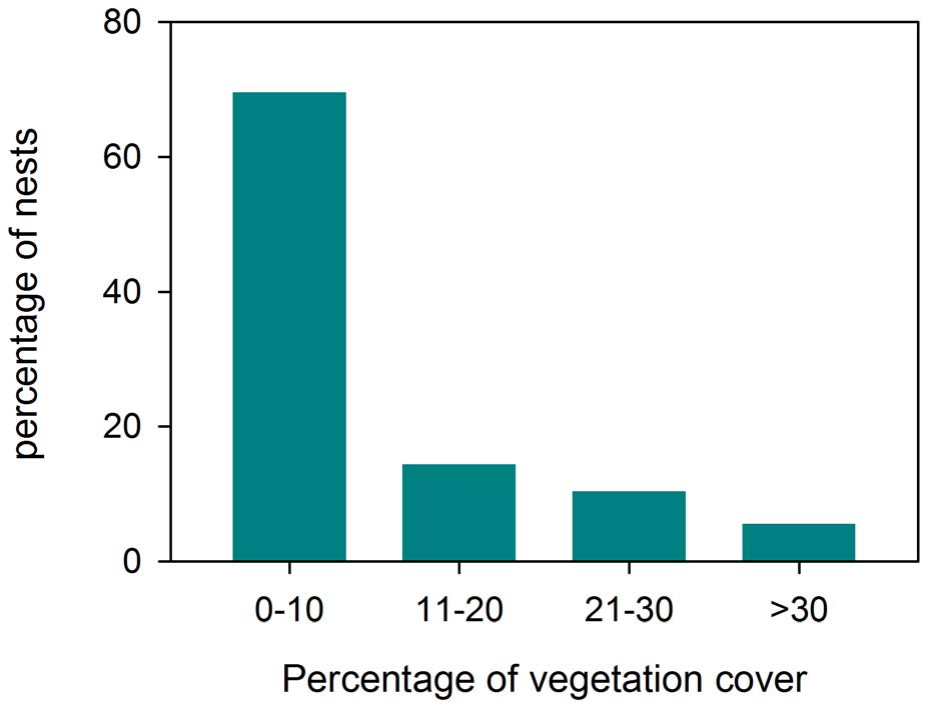
Frequency of Kentish plover nests in relation to vegetation cover. (N = 125).

Vegetation cover was recorded in 125 cases: 39 in Serradal, 19 in Almenara and 67 in Punta. In general, plovers tended to select sites without plants or low vegetation cover to build their nests (Fig. 5). Site choice was not limited by plant cover availability, since plants were abundant in both shifting and semi-fixed dunes in the study sites.

**Figure 6:**
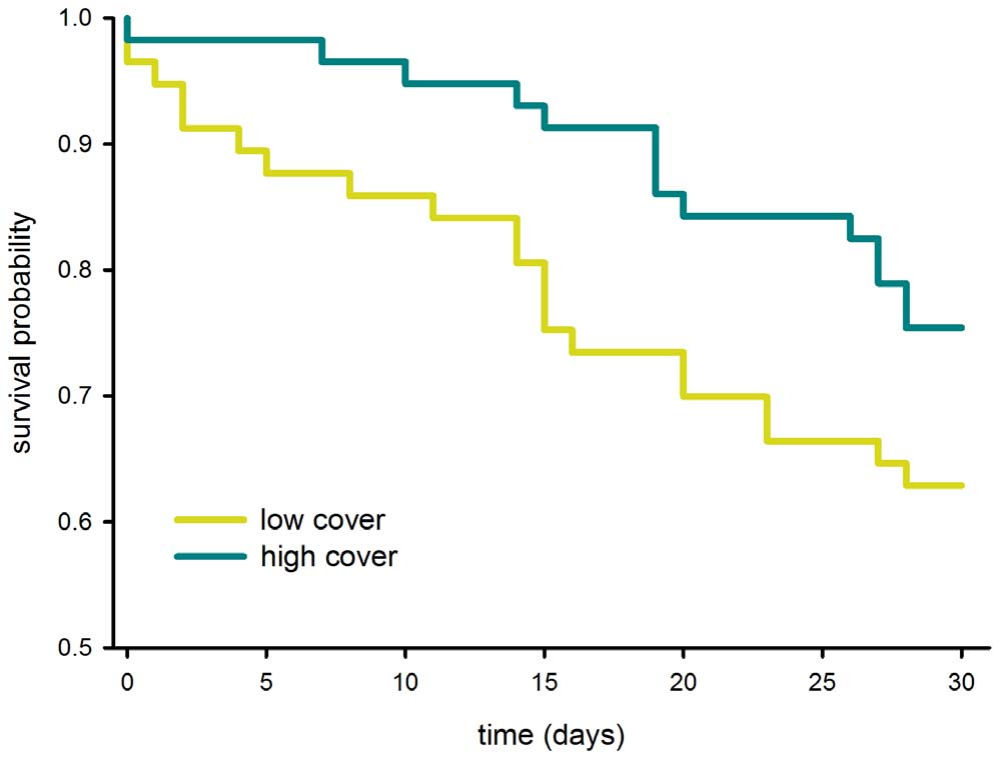
Survival plot from a Cox proportional hazards model with “vegetation cover” fitted as fixed effect. To highlight the effect of vegetation cover on nest survival the original dataset was split into “high” and “low” values of vegetation cover (according to the median) and plotted in two survival plots. Note that the two models represented here do not correspond to the Mixed-effects Cox proportional hazards models shown in Table 1.

Predation and nest desertion were the main causes of clutch failure (predation: 41.4%, desertion: 42.9%, N = 70). Nest survival was affected by vegetation cover (Table 1). A mixed-effects Cox model including habitat type as the only fixed effect did not find a significant effect on survival (Table 1). A model including both variables (cover + habitat) as fixed effects did not provide a better fit than a model including only vegetation cover (*χ^2^* = 6.022, df = 3, P = 0.111). Nests with high vegetation cover showed higher survival probability than nests with low vegetation cover (Fig. 6).

**Table 1:** Mixed-effects Cox proportional hazards models for the survival of Kentish Plover's nests in three beaches located in Eastern Spain.

model	variable	coefficient	exp(coef)	SE(coef)	z	p
cover	vegetation cover	−0.022	0.978	0.009	−2.40	0.017
habitat	habitat (SD)	−0.324	0.723	0.326	−0.99	0.320
	habitat (SFD)	−0.021	0.979	0.297	−0.07	0.940
	habitat (TD)	−0.161	0.852	0.286	−0.56	0.570
cover + habitat	vegetation cover	−0.033	0.967	0.010	−3.31	0.001
	habitat (SD)	−0.562	0.570	0.306	−1.84	0.066
	habitat (SFD)	−0.100	0.905	0.288	−0.35	0.730
	habitat (TD)	−0.567	0.567	0.293	−1.94	0.053

The variable “habitat type” was categorical. All factor levels of this variable were compared with the reference level (i.e., embryonic shifting dunes). Abbreviations: tidal debris (TD), embryonic shifting dunes (ESD), shifting dunes (SD) and semi-fixed dunes (SFD); SE  =  standard error.

### Visibility

The visibility of humans from nests was 3–4 times higher than for dog-sized predators in the beach subject to human disturbance (Serradal, humans: 264±80°, dogs: 68±59°, Wilcoxon Signed Ranks Test, Z = −4,199, P<0.001, N = 23). However, on the undisturbed beach with restricted access to humans, the difference between humans' and dogs' visibility was lower although still significant (Punta, humans: 240±76°, dogs: 181±85°, Wilcoxon Signed Ranks Test, Z = −4,110, P<0.001, N = 22).

**Figure 7:**
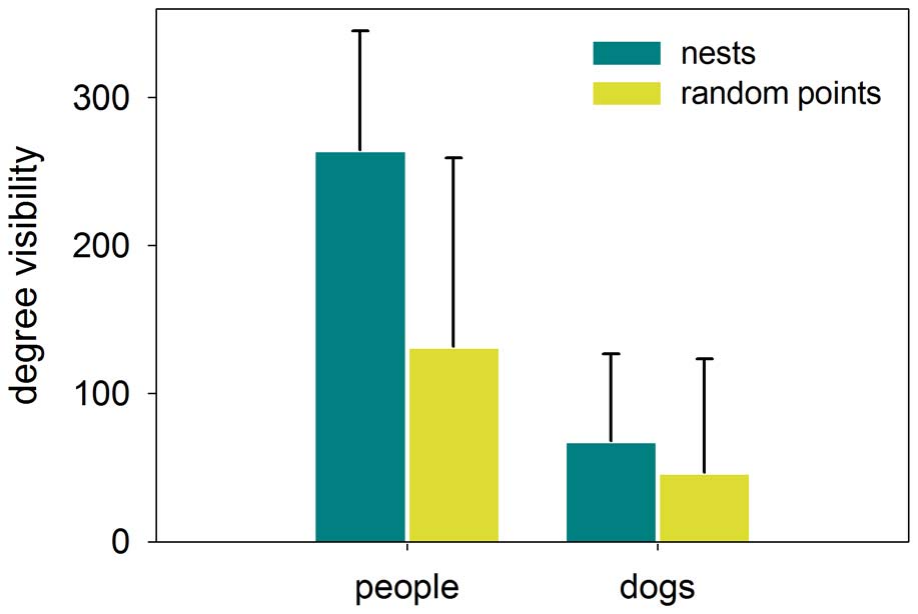
Visibility to potential predators. The comparison of the average visibility towards humans and dog-sized predators from real nest and a set of control points are shown. Error bars indicate the standard deviation.

The visibility from real nests was higher than from control points for both humans and dogs (Mann-Whitney U test, humans: U = 117.0, P = 0.001; dogs: U = 171.0, P = 0.036, N = 46; Fig. 7). With regard to inland and seashore visibility, visibility of humans was higher from real nests than from control points (Mann-Whitney U test, inland: U = 137.5, P = 0.005; seashore: U = 111.0, P<0.001, N = 46). However, there were no differences in the visibility of dogs in both sectors between nests and control points (inland: U = 180.0, P = 0.06; seashore: U = 230.0, P = 0.445, N = 46).

When comparing disturbed and undisturbed beaches (Serradal vs Punta), humans' visibility from nests was similar (Mann-Whitney U test, U = 203.5, P = 0.260), but the dogs' visibility was greater in the beach without human presence (Mann-Whitney U test, U = 71.5, P<0.001). Moreover, in the undisturbed beach (Punta), seashore visibility was higher for both humans and dogs (Wilcoxon Signed Ranks Test, humans: Z = −3.741, P<0.001, dogs: Z = −3.898, P<0.001, N = 22) in comparison with the disturbed beach (Wilcoxon Signed Ranks Tests, humans: Z = 0.865, P = 0.387; dogs: Z = 0.915, P = 0.360; N = 23). Considering beaches together, humans' and dogs' visibility from successful (N = 36) and failed nests (N = 6) was similar (Mann-Whitney U test, humans: U = 98.0, P = 0.719; dogs: U = 95.5, P = 0.653; N = 42).

With regards to nest visibility and vegetation cover, there was a negative relation among them, although not significant (Spearman correlation; humans visibility: *r*_s_ = −0.348, P = 0.113; dogs visibility *r*_s_ = −0.238, P = 0.286; N = 22 in both cases).

### Flushing behavior

Incubating plovers left nests when observers were at a mean distance of 38.7±24.4 m (N = 35). 33 out of 35 cases the incubating adult was the female. The distance at which plovers flushed decreased with vegetation cover (Spearman correlation, *r*_s_ = −0.411, P = 0.020, N = 32). Neither days of incubation nor sand temperature nor time of day affected flushing distance (Spearman correlation: days of incubation, *r_s_* = 0.279, P = 0.110; temperature, *r_s_* = −0.036, P = 0.846; Student's *t* test, time, t_30_ = 0.137, P = 0.892). Flushing distances were higher on the undisturbed beach than the disturbed beach (Serradal, 23.1±12.3 m; Punta, 44.4±25.8 m; Student's *t* test, t_32_ = 2.365, P = 0.024). In 25.7% of the approaches at least one adult (in most cases the female) performed distraction displays towards the observer after nest flushing.

People walking unleashed dogs disturbed more frequently than people walking without dogs (*χ^2^* = 44.977, df = 1, P<0.001). Disturbance caused by dogs resulted in adults flushing from the nest in 73.33% of cases compared with only 14.29% of nest departures caused by humans.

## Discussion

Kentish Plover selected sites without plants or little vegetation cover for nesting, despite the fact that under these conditions nests had a higher failure rate as compared to more sheltered sites. Increased vegetation density and habitat heterogeneity may reduce nest predation rates [43], [44]. Despite this advantage, many shorebirds nest in open habitats typically with very low vegetation cover [8]. In line with this, some studies have shown that Plovers avoid nesting in vegetated areas so as to increase predator detection [2], [13], [14], [45], [46]. Therefore, Plovers show competing interests between adult and nest survival and thus they must balance the benefits of visibility against predation risk when selecting nest-sites [12]. Such evidences have suggested that a trade-off exists between nest crypsis and the ability of incubating adults to detect predators [7], [16]. Our results reveal that Kentish plovers nesting on sandy beaches actively selected nest sites located on the inland part of the beach and on embryonic shifting dunes with little or no vegetation cover. Plovers' nest site selection could be accounted for by two different non-exclusive reasons: (i) to avoid nest flooding during heavy marine storms [47]; and (ii) to minimize adult predation by maximizing the plovers' ability to detect predators [2]. Our results are consistent with previous studies that show that plovers select flat and sparsely vegetated habitats for nesting [11], [13], [21]–[23].

Nest-site selection might be the result of a trade-off between the risk of adult predation and clutch success. Our results reveal a higher success for concealed nests and would therefore support the existence of this trade-off between nest concealment and predator detectability. Furthermore, birds must balance the benefits of thermoregulation against the risk of predation when selecting nest-sites [48]. The trade-off between nest concealment and predator detectability must be solved so as to provide an appropriate microclimate for incubation [2], [49].

Normally plovers use flat or gently sloping sites for nesting [12], [13], [15], [20], [32]. However, even in these situations, the microrelief around the nest may reduce the visibility of the surroundings [50]. Moreover, most studies conducted so far consider that vegetation cover is directly proportional to the degree of predator visibility from the nest [15], [51]. Notwithstanding, this relationship is not always accurate. In fact, our results do not show a significant relationship between vegetation cover and visibility. One of the strengths of our study is that we considered predator detection from the bird's-eye view. This allowed us to distinguish between those elements that constitute a real obstacle to the bird's visual field. For example, a given amount of vegetation may obstruct in a different way the visibility of incubating adults depending on both vision permeability (i.e plant. foliage and branch density) and the distance between the plant and the bird (Fig. 3). Likewise, the absence of vegetation cover should not necessarily be interpreted as a privileged position for predator detection, since elevations in the surroundings of the nest (e.g. the existence of nearby shifting dunes) can substantially reduce adult visibility.

Nesting in open areas increases the detectability of predators but also increases the probability that the incubating adult can be easily detected. However, more conspicuous individuals might be able to compensate for a higher predation risk by modifying their anti-predator behavior [3]. In this context, early flushing behavior may be an effective adaptation against terrestrial nest predators that hunt using olfactory stimuli, because unattended nests are more difficult to find [24]. Animals may adjust their vigilance according to how conspicuous they appear to predators [52]. Early flushing behavior may also be an effective adaptation against nest predators that follow a strategy based on locating nests from the departures of incubating adults [53]. We found that plovers left their nests later when the observer approached with increasing vegetation cover. These results are in agreement with previous studies which found that predation on incubating adults was more frequent at more concealed sites, and that plovers with unrestricted view departed sooner when an observer approached [2].

Once the predator is close to the nest, plovers may perform distraction displays to prevent predators from locating the nest [8], [45]. In the three study areas, plovers frequently performed distraction displays to lure the observer away from nest sites during visits. In fact, nearly 25% of the approximations in the flushing behavior experiment resulted in at least one adult (in most cases the female) performing distraction displays after leaving its nest. This behavior contrasts with that observed in a Kentish Plover population breeding in an inland lake in Spain, where plovers did not perform distraction displays towards humans [2]. Plovers could perform displays towards humans because they consider that humans are potential predators [54]. Interestingly, we found that birds of the undisturbed beach (Punta) behaved similarly to the birds of the other two beaches subject to human disturbance. It is likely that the birds in our study area were more habituated to human presence. However, we observed that plovers left their nests closer to the observer in the beach with high levels of human disturbance than in the undisturbed beach. This suggests that shorebirds breeding in beaches may get used to human presence, and are capable of adjusting anti-predator behavior to disturbance level. Differences in reaction distance suggest that although escape from predation is generally prioritized above other activities [3], including incubation, birds can modulate this behavior when they are habituated to the presence of humans walking [55], [56].

Both humans and dogs are considered predators by shorebirds [31], [51], [57]–[59], and both are directly responsible for a significant number of failed nests [11], [53], [60]. Dogs disturb proportionately more nests than humans on beaches [56] presumably because dogs chase plovers on a regular basis and birds instinctively view them as predators [61]–[63]. On the beach most affected by human presence (Serradal), the main threats of nests and incubating plovers were humans and dogs. Occasionally we observed some beach walkers chasing birds when they performed distraction displays, particularly when birds were simulating to be injured. Furthermore, people sporadically destroyed nests or stole plover eggs (13.56% of nest failures in disturbed beaches). However, disturbance caused by dogs was more frequently recorded than disturbance caused by humans. Domestic dogs were usually walked along the beaches and frequently chased the birds (1.64% of nest failures directly attributed to dogs). We found that visibility from the nests regarding humans was similar on both disturbed and undisturbed beaches. Nevertheless, the view from the nests towards dogs was greater in the undisturbed beach, although the presence of dogs was scarcer. This greater visibility regarding dogs might be explained by the preference of the birds from this beach to locate their nests in open habitats, particularly among the tidal debris, so these sites had better visibility to terrestrial predators. Instead, on the beach most affected by human presence (Serradal) birds tended to locate their nests in sites more distant from seashore. Nesting in less exposed locations might be explained by two non-exclusive reasons. On one hand, plovers would distance from the disturbance caused by bathers and walkers and their pets. On the other hand, birds would be forced to nest on alternative habitats as a result of the beach management, where the tidal debris are periodically removed.

Birds nesting on beaches are under higher level of human disturbance than birds breeding in other habitats [64]. This is particularly important in highly humanized areas, where tourism is one the most important economic activities, such as the Mediterranean coastal areas. The outcome is a progressive narrowing of the suitable breeding habitat for plovers. In disturbed beaches, plovers are forced to move to inland sites. However, inland areas are less suitable because of higher vegetation cover [31], [65]. This constraint on the width of suitable breeding habitat is particularly relevant for the conservation of breeding populations of Kentish Plover, especially under the current context of coastal regression [66], and under future scenarios of sea-level rise from climate change [67], [68]. Therefore, the protection of the widest beaches would be an adequate strategy for plover conservation, given the difficulty of mitigating the effects of coastal erosion. Efforts undertaken so far to reduce the effects of coastal erosion on sandy beaches have been aimed at creating breakwaters and high shifting dunes close to the seashore, typically with high coverage of dune plants. Our results evidence that this type of dune habitat is not the most suitable for the Kentish plover since they avoid sloping areas with high vegetation cover. In fact, this could be one of the causes of many local extinctions or drastic reduction of breeding populations observed along the Mediterranean coast of Spain (authors unpubl. data). We recommend that future dune restorations should take into account Kentish Plover habitat selection [31].
